# Ventral tegmental area amylin / calcitonin receptor signaling suppresses feeding and weight gain in female rats

**DOI:** 10.1016/j.neures.2024.10.001

**Published:** 2024-10-09

**Authors:** Tyler J. Gustafson, Lauren E. McGrath, Matthew R. Hayes, Elizabeth G. Mietlicki-Baase

**Affiliations:** a Department of Exercise and Nutrition Sciences, School of Public Health and Health Professions, University at Buffalo, State University of New York, Buffalo, NY 14214, USA; b Department of Psychiatry, Perelman School of Medicine, University of Pennsylvania, Philadelphia, PA 19104, USA; c Center for Ingestive Behavior Research, University at Buffalo, State University of New York, Buffalo, NY 14260, USA

**Keywords:** Obesity, Mesolimbic, Sex differences, Estrogen, DACRA

## Abstract

The pancreatic peptide amylin promotes negative energy balance in part through activation of amylin receptors (AmyRs) expressed in the ventral tegmental area (VTA), but studies have been limited to male rodents. We evaluated whether VTA amylin signaling governs feeding and body weight in female rats. Indeed, pharmacological VTA AmyR activation suppressed chow intake and body weight in females. Viral-mediated knockdown of VTA calcitonin receptor (GPCR of AmyR) supports the physiological relevance of VTA amylin signaling for energy balance control in females. Collectively, these data support the relevance of VTA amylin signaling for energy balance control in both sexes.

Despite major advances in the development of obesity pharmacotherapies, obesity remains a highly prevalent and costly disease. One hormone system currently under investigation as a potential target for new obesity treatments is amylin (Camilleri and Acosta, 2024), a peptide that is produced in the pancreas and brain. Amylin and amylin receptor (AmyR) agonists act in the brain to promote satiation, thereby suppressing food intake and body weight (Boyle et al., 2022). AmyR consist of a calcitonin receptor (CTR) associated with a receptor activity-modifying protein (Hay et al., 2015), and some ligands activate both AmyR and CTR, which has generated interest in dual amylin/calcitonin receptor agonists (DACRAs) for obesity treatment (Sonne et al., 2021).

The ventral tegmental area (VTA) is an important site of action for AmyR/CTR signaling to suppress feeding. Studies in male rats have shown that VTA AmyR/CTR activation with the DACRA salmon calcitonin (sCT) reduces energy intake and decreases motivation to work for a palatable food (Mietlicki-Baase et al., 2013). However, the paucity of data describing energy balance effects of VTA amylin signaling in females represents a major gap in the literature. Here, we investigate the pharmacological and physiological relevance of VTA AmyR/CTR signaling for feeding and body weight control in adult female rats with normal estrous cycling.

Experiments were approved by the Institutional Animal Care and Use Committee of the University of Pennsylvania or the University at Buffalo. Adult female rats (Charles River) on a 12 h:12 h light:dark cycle were allowed to acclimate to the animal facility before surgery or testing. For testing, rats were individually housed in hanging wire cages in a temperature- and humidity-controlled environment. They had *ad libitum* access to food and water.

First, rats were anesthetized via intramuscular (IM) injection of 90 mg/kg ketamine, 0.64 mg/kg acepromazine, and 2.7 mg/kg xylazine (KAX) and surgically implanted with a bilateral indwelling cannula (Plastics One) aimed at the VTA (guide cannula positioned 6.8 mm posterior to bregma, ±0.5 mm lateral to midline, 6.5 mm ventral to skull; internal cannula aimed −8.5 mm ventral to skull). Analgesia was provided pre-operatively and for 2 days post-operatively (5 mg/kg carprofen, SubQ). After ≥1wk recovery from surgery, rats received unilateral intra-VTA injection of rat amylin (Bachem; 0, 0.1, 0.2 μg) in 100 nl artificial cerebrospinal fluid (aCSF; Harvard Apparatus), using a within-subjects counterbalanced design. Injections were given shortly before lights off, and then pre-weighed chow (Purina 5001) was made available. Food intake was measured at 1, 3, 6, and 24 h post-injection; food spillage was collected and accounted for in all intake measurements. Body weight (BW) was also measured at 0 and 24 h. Then, the same group was used to assess effects of intra-VTA injection of sCT on chow intake and weight gain. Specifically, rats received unilateral injection of sCT (Bachem; 0, 0.01, 0.04 μg; vehicle, 100 nl aCSF) into the opposite VTA hemisphere, again using a within-subjects design. Food intake and BW were measured as described above. After all testing was complete, targeting of injections to the VTA was histologically verified and only rats with cannula terminating within VTA (Paxinos and Watson, 2014) were included in data analyses. Intake and BW data from these experiments were analyzed via repeated measures ANOVA, accounting for the within-subjects factors of drug dose and, where applicable, time. Significant effects were probed with Student-Newman-Keuls posthoc tests.

Next, to assess the physiological relevance of VTA AmyR for feeding and weight gain, a separate group of female rats received adenoassociated viral (AAV)-mediated knockdown of CTR in the VTA to assess the physiological relevance of VTA AmyR/CTR signaling on energy balance control. These rats were maintained either on chow (Teklad 2018, Envigo) or high-fat diet (HFD; Research Diets D12492, 60 % kcal from fat) for ~1.5wk prior to surgery, and then received surgical injection of an shRNA-AAV to knockdown CTR expression (CTR-KD; titer=5.38e12) or an AAV control (CTR-Ctrl; titer=5.33e12), similar to AAV procedures previously described (Mietlicki-Baase et al., 2015). Briefly, rats were anesthetized via IM KAX injection and AAV was bilaterally infused into the VTA (guide cannula positioned 6.8 mm posterior to bregma, ±0.5 mm lateral to midline, 6.5 mm ventral to skull; internal cannula aimed −8.5 mm ventral to skull; 200 nl/hemisphere). We monitored food intake and BW daily for 30 days post-surgery. Then, animals were anesthetized via IM KAX injection and euthanized; brains were collected and flash frozen in isopentane. To confirm effective AAV-mediated VTA CTR-KD, a biopsy punch was used to collect bilateral ~1mm^3^ tissue punches from VTA, which were processed via qPCR (n=5–8/group) to evaluate expression of CTR-A (Thermo Fisher Scientific, Rn01526770_m1), the CTR subtype more highly expressed in VTA than the splice variant CTR-B (Mietlicki-Baase et al., 2013), with GAPDH as the internal control (Thermo Fisher Scientific, Rn01775763_g1).

Food intake data from the AAV-knockdown study were converted to kcal to allow for more accurate comparisons among groups (chow, 3.1 kcal/g; HFD, 5.24 kcal/g). For three instances of technical errors in food intake measurements, the missing data point was replaced by taking the average of that animal’s measurements on the day before and after the missing data. Energy intake and BW data were analyzed via mixed design ANOVA, accounting for the between-subjects factors of AAV and diet, and the within-subjects factor of time. Relative CTR-A mRNA expression data were analyzed by two-way ANOVA with diet and AAV as between-subjects factors. Student-Newman-Keuls posthoc tests were used where appropriate.

Direct administration of amylin to the VTA of female rats (n=6) suppressed chow intake (Fig. 1a). ANOVA revealed main effects of amylin dose (F_2,10_=4.89, p<0.05) and time (F_3,15_=299.63, p<0.05) but no dose-time interaction (F_6,30_=0.95, p=0.47). Posthoc analyses for the dose effect indicated that both doses of VTA amylin significantly reduced chow intake compared to vehicle (both p<0.05). BW gain over 24 h was also reduced by VTA amylin administration (Fig. 1b; F_2,10_=7.22, p<0.05); posthoc tests showed that both doses significantly suppressed BW compared to vehicle-treated animals (p<0.05).

Similar to outcomes produced by VTA injection of amylin, direct VTA injection of sCT in female rats (n=6) decreased chow intake (Fig. 1c). There were significant main effects of sCT dose (F_2,10_=5.87, p<0.05) and time (F_3,15_=155.18, p<0.05) and a significant dose-time interaction (F_6,30_=4.69, p<0.05). Posthoc analyses showed that the higher dose of sCT was significantly different from vehicle at 3 h post-injection, and from both the vehicle and lower sCT dose at 6 and 24 h post-injection. BW change was affected by sCT (Fig. 1d; F_2,10_=11.62, p<0.05), with the higher sCT dose significantly decreasing weight change compared to vehicle or low dose sCT (both p<0.05).

To evaluate the physiological relevance of VTA AmyR signaling for energy balance control in females, we tested the ability of AAV-mediated CTR-KD (versus AAV CTR-Ctrl) to alter feeding and BW in chow-fed and HFD-fed rats, resulting in 4 AAV-diet conditions (n=7–8/group). Effective AAV-mediated knockdown of VTA CTR mRNA expression was confirmed by qPCR (Fig. 2a; main effect of AAV: F_1,22_=20.55, p<0.05; main effect of diet: F_1,22_=0.27, p=0.61; AAV-diet interaction: F_1,22_=2.42, p=0.13). BW on the day of the surgery did not differ among experimental groups (Fig. 2b; no significant effect of AAV, diet, or AAV-diet interaction; all F_1,25_≤3.78, p>0.05). Post-AAV injection, we monitored energy intake and BW for 30 days. Total energy intake (Fig. 2c) and total weight gain (Fig. 2d) post-AAV were significantly higher in HFD-fed versus chow-fed animals (main effects of diet: both F_1,25_≥24.94, p<0.05) and were higher in CTR-KD versus CTR-Ctrl rats (main effects of AAV: both F_1,25_≥6.44, p<0.05). Looking at day-to-day data, daily food intake (Fig. 2e) was affected by both AAV and diet (main effects both F_1,25_≥6.44, p<0.05) as well as time (F29,725=10.13, p<0.05). Time x diet interaction (F29,725=1.65, p<0.05) showed that HFD-fed rats consumed more calories (Days 1–2, 13–30, all p<0.05 versus chow-fed), and time x AAV interaction (F29,725=4.50, p<0.05) demonstrated that CTR-KD rats had higher energy intake near the end of testing (Days 25, 27–28, all p<0.05 versus CTR-Ctrl). For daily BW (Fig. 2f), significant main effects of diet (F_1,25_=17.32, p<0.05) and time (F_29,725_=230.76, p<0.05) were detected, along with Time-AAV and Time-Diet interactions (both F_29,725_≥8.76, p<0.05). SNK posthocs revealed significantly higher BW in HFD-fed compared to chow-fed rats on Days 15–30 (p<0.05) and also in the CTR-KD rats compared to CTR-Ctrl on Days 28–30 (p<0.05).

These findings provide novel evidence supporting the importance of VTA AmyR signaling for energy balance control in female rats. The data from direct VTA injections of AmyR agonists demonstrate that activation of these receptors can reduce both chow intake and weight gain in females, and importantly demonstrate that both the native ligand of rat amylin, as well as the pharmacological agonist sCT that activates AmyR and CTR, exert similar directions of effect on these outcomes. The sCT doses used here were selected based on similar studies in male rats (Mietlicki-Baase et al., 2013, Mietlicki-Baase et al., 2017) and suggest that the effects of these doses are relatively comparable between sexes, at least for effects on chow intake; future studies should also examine effects on HFD intake. The doses of amylin are lower than those used in male rats in recent VTA injection studies (Geisler et al., 2024), suggesting the importance of a more comprehensive examination of effective dose ranges in both sexes.

The current CTR-KD study further demonstrates physiological relevance of VTA AmyR for control of feeding and BW in females. Although VTA CTR-KD promoted positive energy balance in female rats, interestingly, no AAV-diet interaction was detected, suggesting that CTR-KD is able to modulate feeding and BW in females regardless of diet. This is intriguing because a prior study in males showed that VTA CTR-KD produced hyperphagia and weight gain in HFD-fed animals, but had minimal effects in chow-fed rats (Mietlicki-Baase et al., 2015). To date, it is unclear what the endogenous source(s) of amylin are that provide input to VTA AmyR. Amylin is able to cross the blood-brain barrier (Banks et al., 1995, Banks and Kastin, 1998), suggesting that pancreatic amylin could potentially reach these receptors. However, another possibility is that amylin-producing cells in the brain may represent central sources of this peptide. Amylin has been detected in various sites in rodent brain including the bed nucleus of the stria terminalis and hypothalamus (Dobolyi, 2009, Szabo et al., 2012, Li et al., 2015), but the target receptor populations for these amylin sources remain unresolved. This is particularly intriguing in the context of sex differences in amylin responses because central production of amylin can be influenced by sex as well as other factors including maternal behavior, social interaction, and diet (Szabo et al., 2012, Li et al., 2015, Fukumitsu et al., 2022), suggesting that the differences in amylin-mediated energy balance between males and females could be due to differential production of amylin at these sites or other nuclei.

These studies add to a small but growing body of literature demonstrating the influence of biological sex in amylin-mediated energy balance control. In addition to sex-divergent central amylin expression which might in turn affect energy balance, it has also been shown that estrogens in females alter the ability of amylin to suppress feeding. Acutely, estradiol enhances the hypophagic effects of amylin during the estrous cycle (Asarian and Geary, 2013, Asarian et al., 2011). In contrast, estradiol can blunt the effects of chronic amylin on feeding in ovariectomized rats (Trevaskis et al., 2010), suggesting that the ways in which amylin interacts with chronic versus acute estradiol levels may differ. The female rats in our study were intact, but estrous cycle was not tracked as we intended to establish whether VTA AmyR signaling in females had any effect on energy balance control. It will be important in future work to evaluate the effects of VTA amylin signaling on feeding in the context of the different stages of the estrous cycle, as well as to establish whether longer-term interactions with ovarian hormones also influence the energy balance effects of VTA AmyR activation.

To our knowledge, these studies provide the first evidence that VTA AmyR signaling in females controls energy balance. These findings build upon prior findings in male rats highlighting the importance of VTA AmyR for energy balance control, but also suggest that there could be differences in how VTA AmyR are engaged by endogenous ligands in females compared to previous findings in male rats. The current findings highlight VTA AmyR signaling as an important mediator of energy balance control in both sexes, and underscore the need for deeper investigation of sex differences in mesolimbic amylin signaling.

## Figures and Tables

**Fig. 1.**
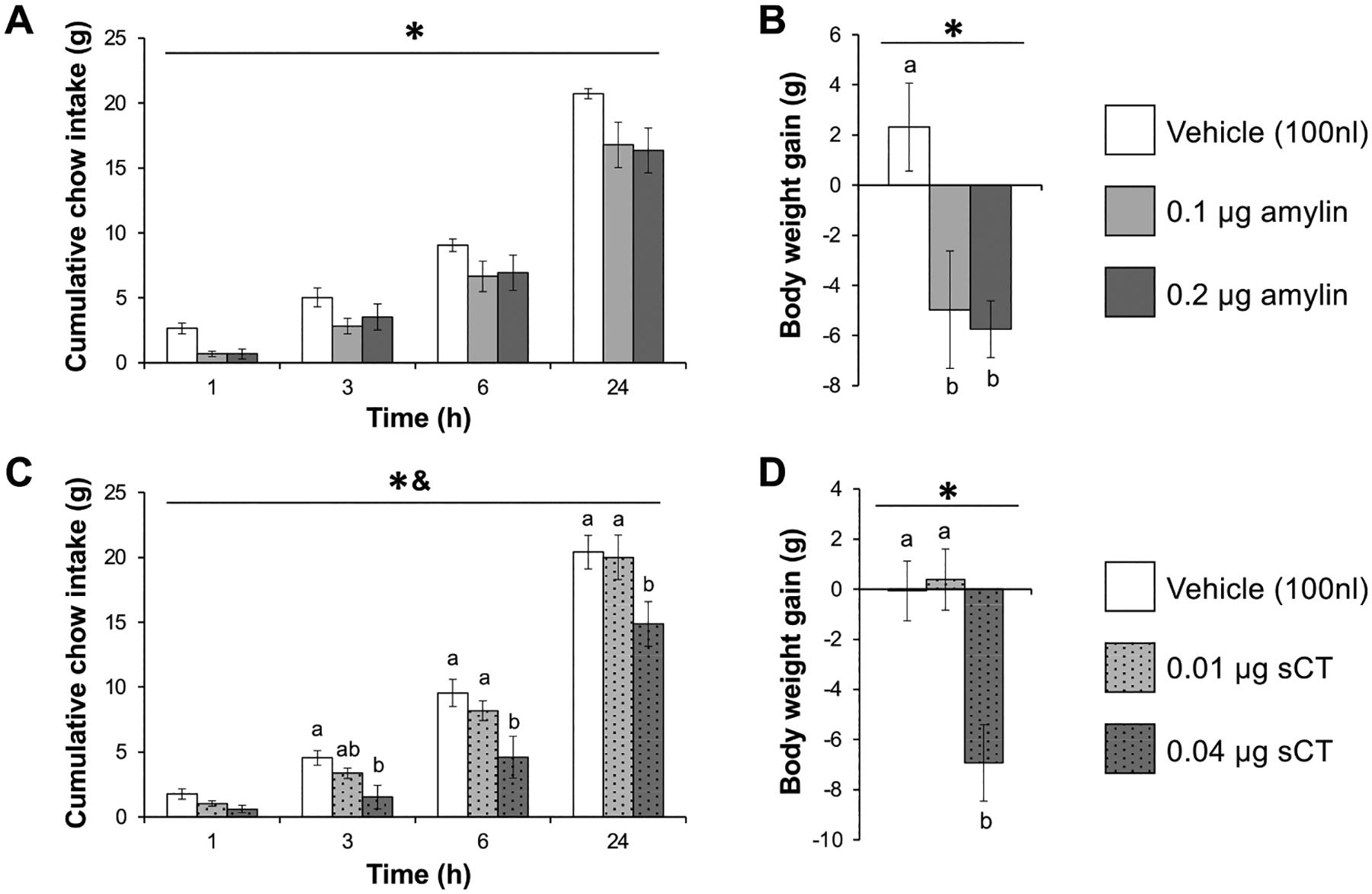
Pharmacological activation of VTA AmyR reduces food intake and body weight gain in female rats. After direct VTA injection of amylin in females (n=6), both doses tested significantly suppressed cumulative chow intake (A) and body weight gain (B) in the 24 h post-injection. Key in B applies to panels A and B. Intra-VTA sCT at the higher dose tested reduced cumulative chow intake (C) beginning at 3 h post-injection and lasting through the rest of the 24 h test period, and also reduced body weight gain (D) in female rats (n=6). Key in D applies to panels C and D. Data are shown as mean ± SEM. *, main effect of dose (p<0.05); &, dose-time interaction (p<0.05). Different letters above bars represent significant differences between groups (p<0.05) for the dose-time interaction for food intake, and for the dose effect for body weight gain.

**Fig. 2.**
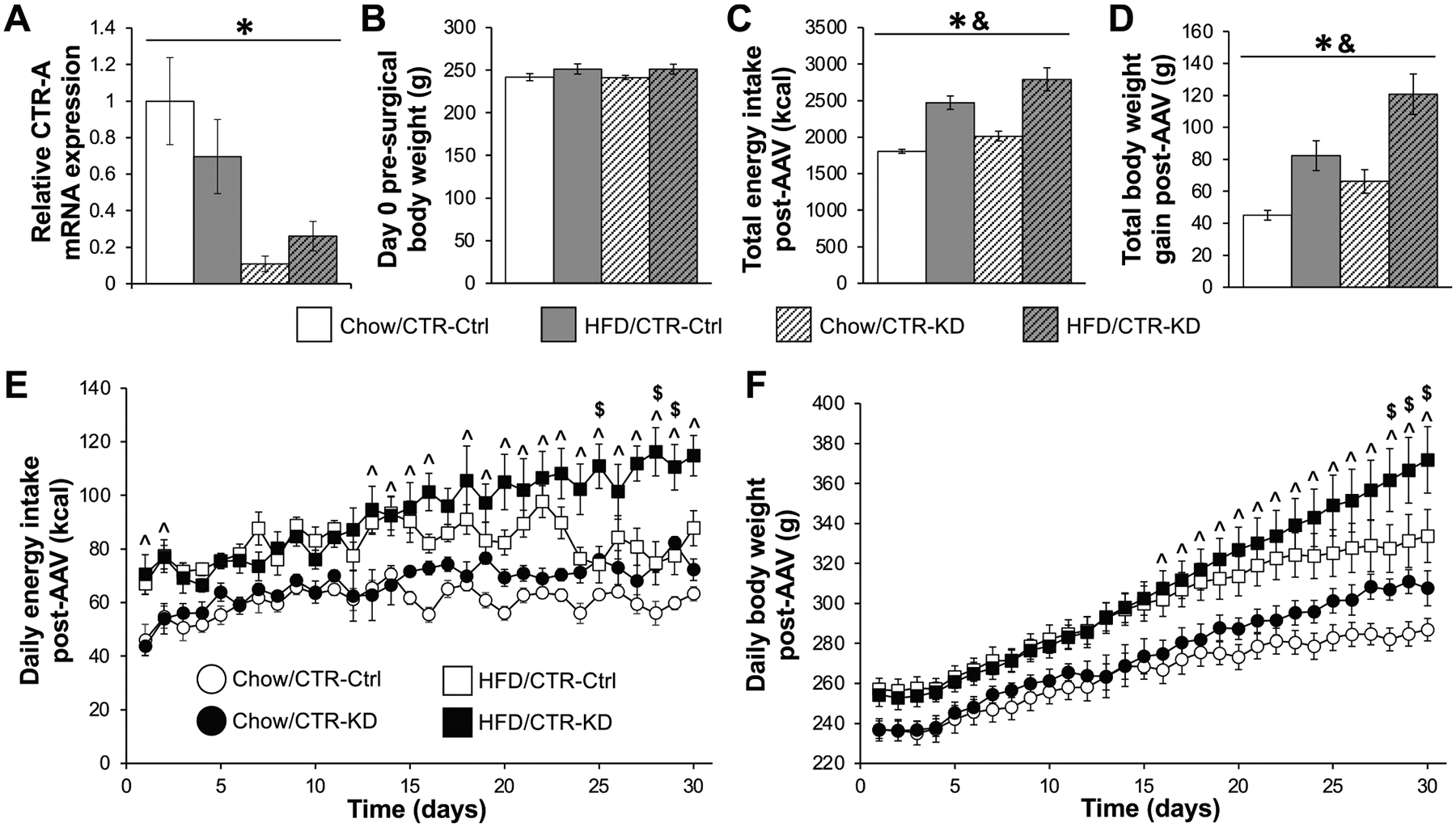
AAV-mediated knockdown of VTA CTR/AmyR increases feeding and weight gain in female rats. VTA CTR knockdown was confirmed via qPCR (A). There were no differences in body weight among groups on the day of AAV injection (B). Total energy intake (C) and total body weight gain (D) post-AAV were increased in CTR-KD groups compared to CTR-Ctrl. Daily energy intake (E) and daily body weight (F) were higher in HFD-fed compared to chow-fed rats during much of the testing period, and higher in CTR-KD versus CTR-Ctrl towards the end of testing. Key in E applies to panels E and F. Data are shown as mean ± SEM. For feeding and body weight data, n=7–8/group; for qPCR, n=5–8/group. *, main effect of AAV (p<0.05); &, main effect of diet (p<0.05); ^, diet-time interaction (chow versus HFD posthoc analyses, p<0.05); $, AAV-time interaction (CTR-Ctrl versus CTR-KD posthoc analyses, p<0.05). Different letters above bars represent significant differences between groups (p<0.05) for the dose-time interaction for food intake, and for the dose effect for body weight gain.
